# Effect of Strong Electric Fields on Material Responses: The Bloch Oscillation Resonance in High Field Conductivities

**DOI:** 10.3390/ma13051070

**Published:** 2020-02-28

**Authors:** Satyvir Singh, Marco Battiato

**Affiliations:** Division of Physical and Applied Physics, School of Physical and Mathematical Sciences, Nanyang Technologica University, 21 Nanyang Link, Singapore 637371, Singapore; marco.battiato@ntu.edu.sg

**Keywords:** Boltzmann transport, modal discontinuous Galerkin, Bloch oscillation resonance, strongly out-of-equilibrium, conductivity

## Abstract

In this paper, we investigated the effect of strong electric fields on material responses and the Bloch oscillation resonance in high field conductivities. For this purpose, a high-order accurate explicit modal discontinuous Galerkin (DG) solver is employed for solving the quantum Boltzmann transport equation (BTE) in the context of electron transport at nanoscales under strongly out-of-equilibrium conditions. Here, we study the transient behavior and the convergence of a steady-state response to an external oscillating electric field switched on at time zero. We first benchmark our numerical results with known analytic steady-state responses at low fields. The computational results show that the present DG scheme is in excellent agreement with analytic solutions over the whole range of parameters and to an extremely high precision, allowing us to achieve good agreement even for the fifth-order response at low fields. We then extend the method to strong electric fields and show how the responses are deviated from the low-field ones and the transition to a dampened Bloch oscillation regime. Most importantly, we report the observation of a new regime induced by the resonance between the standard low-field response and Bloch oscillations.

## 1. Introduction

Transport phenomena in solids are the focus of an extremely wide range of research topics. The transport of quasi-particles (for example, electrons, phonons, or more complex quasiparticles like excitons) in nanotechnology, plasmonics, quantum computer technology, and microelectromechanical systems (MEMS) is of great importance due to their tremendous technological and scientific applications [1,2,3]. Transport phenomena are intrinsically out-of-equilibrium. However, until recently, for most applications, treating near equilibrium configurations has been sufficient. The rise of the field of ultrafast dynamics has brought forward the necessity of going beyond common approximations used close to equilibrium [4,5,6,7,8,9,10,11,12]. However, this importantly increases the complexity of the treatment as a large degrees of freedom value is no longer constrained. The unfreezing of these degrees of freedom becomes critical as qualitatively new phenomena emerge from the ensuing complexity. It therefore becomes essential to develop theoretical approaches that can fully handle the arising complexity. In the early years of the semiconductor industry, macroscopic models such as the drift-diffusion model or the hydrodynamic model have been sufficient for device simulation. Conversely, accurate simulations of modern nanoscale devices within the field of ultrafast dynamics require the use of more precise models.

The quantum Boltzmann equation has been widely proven to have the power and flexibility to describe a very wide range of dynamics of quasi-particle excitations in solids. It allows researchers to describe transport and thermalization even in heterogeneous systems. However, its application has been limited to approximated cases. The most famous is the calculation of conductivities in uniform systems, as a perturbation of the equilibrium state. However, when solved far from equilibrium, the Boltzmann equation becomes quickly numerically challenging. Here, we apply a relatively new numerical approach, discontinuous Galerkin, to the Boltzmann equation. Such method has been shown to have a number of technical and numerical advantages, such as hp-adaptivity, conservative, stable, robust with strong mathematical supports, weak approximation of boundary conditions, and built-in parallelism which permits coarse-grain parallelization [13,14,15,16,17,18]. Here, we first analyze its performance on physically relevant quantities and highlight the deep insight it provides. Critically, we also show that the method allows for the accurate description and prediction of strongly-out-of-equilibrium dynamics without compromising its precision close to equilibrium: we show that it achieves an extremely high precision in constructing high-order low-field responses.

Finally, we use the method to highlight the evolution of the responses as the system moves further from equilibrium. We show how the material response can be analyzed in terms of conductivity and nonlinear conductivities even in the presence of increasing electric fields. We show how the responses are altered further from equilibrium. The key finding is that a clear electric field amplitude-dependent resonance appears in the conductivity and nonlinear conductivities. We identify its origin as arising from the resonance between a more standard low-field driven response and dampened Bloch oscillations.

## 2. Boltzmann Transport Equation for Electrons

The time evolution of quasi-particle excitations in materials is described by a distribution function f(x,k,t), where $x\in {ℜ}^{3}$ is the position in real space, $k\in {ℜ}^{3}$ is the momentum vector, and t>0 is the time. The domain of k is the first Brillouin zone (BZ). This distribution function fulfills the Boltzmann transport equation (BTE) [19,20],

(1) $$\frac{\partial f}{\partial t}+v_{x}\cdot \frac{\partial f}{\partial x}+v_{k}\cdot \frac{\partial f}{\partial k}={\left(\frac{\partial f}{\partial t}\right)}_{coll},$$

where ${\left(\frac{\partial f}{\partial t}\right)}_{coll}$ represents the Boltzmann collision integral of the interaction between two quasiparticles. With the prescribed initial condition $f\left(x,k,0\right)=f_{0}\left(x,k\right)$, Equation (1) must be solved for phase space coordinates $\left(x,k\right)\in \Omega_{x}\times \Omega_{k}=\Omega$ and time $t\in \left[0,t_{f}\right]$. The quantities $v_{x}$ and $v_{k}$ appearing in Equation (1) represent the velocities in real and momentum spaces (due to external forces) which are derived from single-particle Schrodinger equation and given as

(2) $$\begin{array}{cc} v_{x} & =\frac{1}{\hbar }\nabla_{k}\epsilon \left(k\right),\\ v_{k} & =\frac{-e}{\hbar }\left(E\left(x,t\right)+v_{x}\left(k\right)\times B\left(x,t\right)\right), \end{array}$$

where the contributions of anomalous velocity is neglected [21]. Here, *e* is the absolute value of electron charge, *ℏ* is the reduced Planck constant, ϵ(k) is the energy dispersion relation, E(x,t) is the macroscopic electric field, and B(x,t) is the magnetic field. The right-hand side of Equation (1) is the collision term, or collision operator, representing all the scattering processes of the electrons.

In the present study, we want to (a) benchmark the numerical method for physically relevant observables and (b) observe the behaviour of the system as it is driven progressively further and further from equilibrium. For this purpose, we address only the one-dimensional BTE in the absence of magnetic field, i.e., $\frac{\partial f}{\partial x}=0$, and B=0. Then,

(3) $$\frac{\partial f}{\partial t}-\frac{e}{\hbar }E\frac{\partial f}{\partial k}={\left(\frac{\partial f}{\partial t}\right)}_{coll}\;\forall \left(k,t\right)\in \Omega_{k}\times \left[0,t_{f}\right].$$

In the present work, we are concerned with testing the capability of the numerical technique to produce accurate predictions for transport properties and investigate the effect of strongly out-of-equilibrium configurations on transport. To be able to have analytical benchmarks close to equilibrium, we adopt a simple collisional model: the so-called relaxation time approximation (RTA) for the collisional operator which is defined as

(4) $${\left(\frac{\partial f}{\partial t}\right)}_{coll}=-\frac{f\left(k,t\right)-f_{eq}\left(k\right)}{\tau }.$$

Here, $f_{eq}\left(k\right)$ is a static distribution function which describes a local equilibrium, while the parameter τ is the relaxation time which characterizes the speed of recovery of the equilibrium state. For electrons, the $f_{eq}\left(k\right)$ is the Fermi-Dirac distribution defined as

(5) $$f_{eq}\left(k\right)=\frac{1}{1+e^{\beta (\epsilon (k)-\mu )}},$$

with $\beta =1/k_{B}T$ being the inverse temperature, $k_{B}$ the Boltzmann constant, *T* the absolute temperature, and μ the chemical potential. Concluding, the BTE (1) reduces to

(6) $$\frac{\partial f}{\partial t}-\frac{e}{\hbar }E\frac{\partial f}{\partial k}=-\frac{f\left(k,t\right)-f_{eq}\left(k\right)}{\tau }.$$

In the present study, the electric field E is considered a time-dependent function defined as $E\left(t\right)=E_{0}\sin\left(\omega t\right)$, where $E_{0}$ and ω are the amplitude and frequency of the electric field, respectively. The electric field is switched on at time t=0, triggering a transient behavior, before the system reaches a steady state.

**Figure 1:**
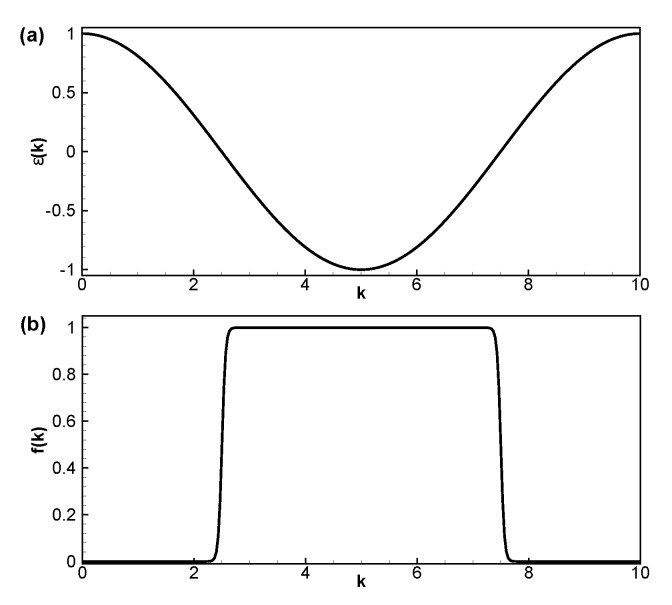
(**a**) Diagram of the used energy dispersion relation in the first Brillouin zone. (**b**) Example of momentum-resolved population at thermal equilibrium.

For the numerical simulations, we consider a simple tight-binding dispersion relation, ϵ(k)=cos(2πk), $\forall k\in \Omega_{k}=\left[0,L\right]$ which is shown in Figure 1a. The numerical method has the full flexibility to handle a generic band structure; however, we opted to analyze this simple case, since it is familiar to the vast majority of the readers and yet it allows for the appearance of a number of nontrivial effects compared, for instance, to a parabolic band structure. The associated Fermi-Dirac distribution function is plotted in Figure 1b.

## 3. Numerical Method Based on Modal Discontinuous Galerkin Approach

In order to discretize Equation (6), the domain $\Omega_{k}=\left[0,L\right]$ is partitioned into *N* uniform and nonoverlapping elements as follows:

(7) $$0=k_{1/2}<k_{3/2}<\cdots <k_{N+1/2}=L.$$

The grid is defined by $I_{n}=\left[k_{n-1/2},k_{n+1/2}\right],\;\forall n\in 1,2,\cdots N$, where $k_{n}=\frac{1}{2}\left(k_{n-1/2}+k_{n+1/2}\right)$ is the center point and $\Delta k=k_{n+1/2}-k_{n-1/2}$ denotes the mesh size of the element $I_{n}$. For the domain $\Omega_{k}$, we introduce the piecewise polynomial space of the functions $v:\Omega_{k}⟼ℜ$ as

(8) $$V_{h}^{l}=\left\{v\in L_{2}\left(\Omega_{k}\right),v\;|{\;}_{\Omega_{k}}\in P^{l}\left(I_{n}\right),\;\forall n\in 1,2,\cdots N\right\},$$

where $L_{2}\left(\Omega_{k}\right)$ is the Lebesgue space of square integrable functions over the domain $\Omega_{k}$ and $P^{l}\left(I_{n}\right)$ is the space of polynomial functions of degree at most *l* in element $I_{n}$. The numerical solution of Equation (6) is approximated by $f_{h}\in V_{h}^{l}$ in the local element $I_{n}$,

(9) $$f_{h}\left(k,t\right)=\sum_{i=1}^{N_{p}}\hat{f_{h}^{i}}\left(t\right)\varphi_{i}\left(k\right),\;k\in I_{n},$$

where $\hat{f_{h}^{i}}$ is the local degree of freedom and $\varphi_{i}\left(k\right)$ is the basis function. The number of basis functions $N_{p}$ depends on the order of approximation *l* and is given by $N_{p}=l+1$. In the present study, the orthogonal scaled Legendre (modal) basis function is used for function $\varphi_{i}\left(k\right)$ in the reference element $I_{n}=\left[-1,1\right]$.

(10) $$\begin{array}{cc} \varphi_{n}\left(\xi \right) & =\frac{2^{n}{\left(n!\right)}^{2}}{(2n)!}P_{n}^{0,0}\left(\xi \right),\;0\leq n\leq l,\\ \xi & =\frac{2(k-k_{j})}{\Delta k},\;-1\leq \xi \leq 1, \end{array}$$

where $P_{n}^{0,0}\left(\xi \right)$ is the Legendre polynomial and $k_{j}$ is the centre of element. Subsequently, the BTE (6) is multiplied with the test function, which is taken to be equal to the basis function $\varphi_{n}\left(k\right)$, and then integrated by parts over an element $I_{n}$. This results in the following weak formulation of Equation (6) for $f_{h}$,

(11) $$\begin{array}{c} \frac{\partial }{\partial t}\int_{I_{n}}f_{h}\varphi_{h}dk-\int_{I_{n}}\nabla_{k}\varphi_{h}\cdot F\left(f_{h}\right)dk\\ +\int_{\partial I_{n}}n\cdot F\left(f_{h}\right)\varphi_{h}dk=\int_{I_{n}}S\left(f_{h}\right)\varphi_{h}dk, \end{array}$$

where $F=-\frac{e}{\hbar }Ef$, $S=-\frac{f-f_{eq}}{\tau }$. The $\partial I_{n}$ denotes the boundaries of the element $I_{n}$ and n is the outward unit normal vector. The flux function n·F appearing in Equation (11) is substituted by a numerical flux function. Here, the local Lax-Friedrichs flux ${\hat{F}}^{LLF}$ is considered [22]:

(12) $$n\cdot F\equiv {\hat{F}}^{LLF}\left(f_{h}^{-},f_{h}^{+}\right)=\frac{1}{2}\left[F\left(f_{h}^{-}\right)+F\left(f_{h}^{+}\right)-C\left(f_{h}^{-}+f_{h}^{+}\right)\right],$$

where

$$\begin{array}{c} C=max|F^{{}^{\prime }}\left(s\right)|,\;min\left(f_{h}^{-},f_{h}^{+}\right)\leq s\leq max\left(f_{h}^{-},f_{h}^{+}\right). \end{array}$$

Here, the superscripts (+) and (−) respectively denote the inside and outside of an elemental interface.

Several numerical techniques—such as Newton-Cotes, Monte Carlo, trapezoidal, quadrature, etc.—are available in literature [14,23] to compute the integrals appearing in Equation (11). Among them, the quadrature theory is widely used in the discontinuous Galerkin (DG) community because of several desirable properties such as positivity of weights and symmetry. Quadrature methods are also optimal for integrating polynomials in one dimension. In the present study, the volume and boundary integrations appearing in Equation (11) are computed by Gauss-Legendre quadrature rule with (2l+1) quadrature points to ensure accuracy [14,15].

By assembling all of the elemental contributions together, the semi-discrete DG formulation for the BTE system (6) yields an ordinary differential equation (ODE) in time for each element as

(13) $$\frac{df_{h}}{dt}=M^{-1}L\left(f_{h}\right).$$

Here, $M^{-1}$ is the inverse of orthogonal mass matrix and $L(f_{h})$ is the residual function. In our present work, an explicit time scheme of the solution is performed with high-order strong-stability-preserving (SSP) Runge-Kutta methods that preserve the monotonicity of the spatial discretization in any norm or seminorm coupled with first-order forward Euler time stepping [24]. The explicit third-order accurate SSP Runge-Kutta method proposed by Shu and Osher [25] is employed,

(14) $$\begin{array}{cc} f_{h}^{\left(1\right)} & =f_{h}^{n}+\Delta tM^{-1}L\left(f_{h}^{n}\right)\\ f_{h}^{\left(2\right)} & =\frac{3}{4}f_{h}^{n}+\frac{1}{4}f_{h}^{\left(1\right)}+\frac{1}{4}\Delta tM^{-1}L\left(f_{h}^{\left(1\right)}\right)\\ f_{h}^{n+1} & =\frac{1}{3}f_{h}^{n}+\frac{2}{3}f_{h}^{\left(2\right)}+\frac{2}{3}\Delta tM^{-1}L\left(f_{h}^{\left(2\right)}\right), \end{array}$$

where $L(f_{h}^{n})$ represents a numerical approximation of the solution at time $t_{n}$, and the time step Δt is chosen according to the following relation [15]:

$$\begin{array}{c} \Delta t\leq \frac{CFL}{\left(2l+1\right)}\frac{\Delta k}{|\frac{e}{\hbar }E|}. \end{array}$$

Here, CFL is the Courant-Friedrichs-Lewy condition (CFL≤1).

## 4. Analytical Solution for the Steady-State BTE

In this section, for the sake of completeness, we report the analytical solution for the steady-state BTE response in the low-field regime. We will use this regime to benchmark our numerical results. In case of small electric field, the BTE with the relaxation time approximation (6) can be expanded in orders of the perturbation. Neglecting the electron’s transient behavior, the BTE solution at low electric field can be written as the imaginary part of

(15) $$\begin{array}{c} f\left(k,t\right)\approx f_{0}\left(k\right)+E_{0}\delta f_{1}\left(k\right)e^{i\omega t}+E_{0}^{2}\delta f_{2}\left(k\right)e^{2i\omega t}+E_{0}^{3}\delta f_{3}\left(k\right)e^{3i\omega t}+\cdots , \end{array}$$

where ω is the electric field frequency and each term at a generic order *N* can be calculated as

(16) $$\begin{array}{cc} f_{0}\left(k\right) & =f_{eq}\left(k\right),\\ \delta f_{N}\left(k\right) & ={\left(\frac{e\tau }{\hbar }\right)}^{N}\frac{d^{N}f_{eq}\left(k\right)/dk^{N}}{\prod_{n=1}^{N}\left(1+in\omega t\right)}. \end{array}$$

When the BTE dynamics are known, important collective quantities, such as the current density, can be calculated as

(17) $$J\left(t\right)=-\frac{e}{2\pi \hbar }\int_{\Omega_{k}}\nabla_{k}\epsilon \left(k\right)f\left(k,t\right)dk.$$

From Equation (17), one can obtain the current at the appropriate frequency and consequently the complex conductivity as well as all the nonlinear corrections to it (in the presence of time reversal symmetry, the even order currents vanish),

(18) $$\begin{array}{cc} \sigma & =-\frac{e^{2}\tau }{2\pi \hbar^{2}}\frac{\int_{0}^{L}\nabla_{k}\epsilon \left(k\right)\frac{df_{eq}\left(k\right)}{dk}}{\left(1+i\omega \tau \right)},\\ \sigma_{3} & =-\frac{e^{4}\tau^{3}}{2\pi \hbar^{4}}\frac{\int_{0}^{L}\nabla_{k}\epsilon \left(k\right)\frac{d^{3}f_{eq}\left(k\right)}{dk^{3}}}{\left(1+i\omega \tau )(1+2i\omega \tau )(1+3i\omega \tau \right)},\\ \sigma_{N} & =-\frac{e^{N+1}\tau^{N}}{2\pi \hbar^{N+1}}\frac{\int_{0}^{L}\nabla_{k}\epsilon \left(k\right)\frac{d^{N}f_{eq}\left(k\right)}{dk^{N}}}{\Pi_{n=1}^{N}\left(1+ni\omega \tau \right)}. \end{array}$$

Thus, the current from Equation (18) can be expressed as

(19) $$J\left(t\right)\approx \sigma E_{0}e^{i\omega t}+\sigma_{3}E_{0}^{3}e^{3i\omega t}+\sigma_{5}E_{0}^{5}e^{5i\omega t}+\cdots$$

## 5. Convergence Study and Validation of Numerical Solver

### 5.1. Convergence Study

In order to verify the code and estimate the order of accuracy of the numerical scheme, we first solve a simple problem for which an analytical solution exists. Here, we examine the accuracy of the present numerical scheme with $P^{1},P^{2},P^{3}$, and $P^{4}$ elements. The $L^{2}$ and $L^{\infty }$ errors are measured in terms of the discrete norms by

(20) $$\begin{array}{cc} L^{\infty }-error & =max(|\hat{f}\left(k,t\right)-f_{h}\left(k,t\right)|),\\ L^{2}-error & =\sqrt{\sum_{I_{n}}\int_{I_{n}}|\hat{f}\left(k,t\right)-f_{h}\left(k,t\right){|}^{2}}dk,\\ Order & =\frac{log(error\left(N_{1}\right)/error\left(N_{2}\right))}{log(N_{2}/N_{1})}, \end{array}$$

where $\hat{f}\left(k,t\right)$ is the exact solution and $f_{h}\left(k,t\right)$ is the approximate solution. The $error(N_{1})$ and $error(N_{2})$ are the errors (for $L^{2}$, and $L^{\infty }$) for the numbers of elements *N* and 2N, respectively. Our convergence study was done for the following initial value problem,

(21) $$\begin{array}{cc} \frac{\partial f}{\partial t}+\frac{\partial f}{\partial k} & =0,\;k\in [0,2\pi ],\\ f(k,0) & =\sin k, \end{array}$$

with the periodic boundary condition f(0,t)=f(2π,t). The exact solution of this problem is given by f(k,t)=sin(k−t). This problem was calculated up to 4th-order of accuracy at time $t_{f}=1$ by varying the number of grid points N(20,40,80,160). The results of the convergence study with the order of accuracy $L^{2}$ and $L^{\infty }$ are illustrated in Table 1. From the results, it was confirmed that the present numerical scheme achieved the desired order of accuracy (l+1).

### 5.2. Validation of Numerical Solver

**Figure 2:**
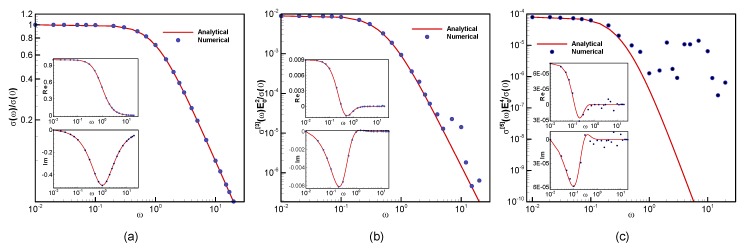
Validation of numerical solver: comparison between numerical and analytical results for calculated (**a**) first-order, (**b**) third-order, and (**c**) fifth-order conductivity at $E_{0}=2\times 10^{3}$ V/cm, τ=1.0, β=50, and μ=−0.8.

An analytic solution cannot be evaluated when the full BTE with a time-dependent external field and the relaxation time approximation term is used. In order to verify the reliability and accuracy of the present numerical solver, we compute the different orders of electrical conductivity for small electric fields with the low-field analytical expressions for the high-order conductivities obtained at the steady state, as discussed in Section 4. We let the simulations run long enough for the steady state to be reached and then apply the Fourier transform to the current temporal profile over the last period. We then compare the Fourier components. We computed the numerical and analytical solutions for electric field, $E_{0}=2\times 10^{2}$ V/cm and at β=50, τ=1.0, μ=−0.8, with a wide range of frequency value ω=0.01−20. Figure 2 shows the numerical and analytical conductivity for first-, third-, and fifth-order (higher order responses have been multiplied by a power of the amplitude of the electric field to have the same dimensionality of a conductivity, and then all the responses were normalized to the zero frequency conductivity for a more direct comparison). The computed results for real, imaginary, and magnitude parts of conductivity are found to be in excellent agreement with the analytical solutions, even for the high-order responses. Discrepancy arises when the response becomes too small compared to the numerical error. This shows how this method, that describes the population within the full Brillouin zone, can achieve an extremely high precision, even in the low-field regime, where the absolute modification of the population is extremely small and restricted to very limited momentum regions.

**Figure 3:**
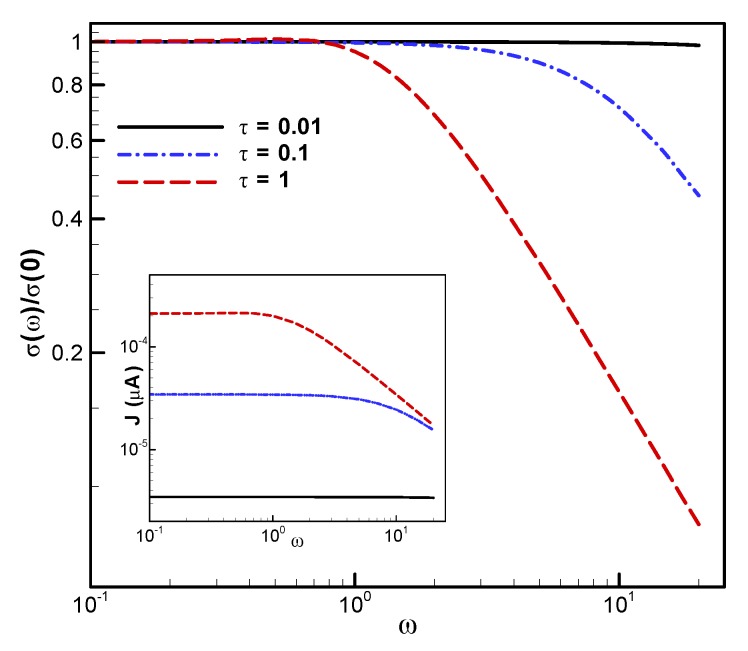
Computed electrical conductivity for three different relaxation times τ=0.01,0.1, and 1 at $E_{0}=10^{4}$ V/cm, β=50, and μ=−0.8.

Finally, in Figure 3, we show the dependence of the conductivity on the relaxation time. Consistently with Equation (18), the relaxation time controls both the amplitude of the conductivity and the frequency at which the drop in conductivity is observed.

## 6. Numerical Results and Discussion

In this section, we present the numerical results for the BTE dynamics in regimes that range from equilibrium to strongly nonequilibrium. Emphasis is placed on the time evolution of the distribution function, including the effects of physical parameters and steady-state electrical conductivity. In this work, we express the parameters in the following units: volts per centimeter (V/cm) for the electric field; one over nanometer (1/nm) for the momentum; picosecond $(=10^{-12}$ s) for the time; Terahertz $\left(=10^{12}hertz\right)$ for the frequency; and picosecond $(=10^{-12}$ s) for the relaxation time. The constant value of the ratio e/ℏ is $1.54\times 10^{-4}$. In general, the solutions of the BTE system are largely affected by the following flow parameters: the electric fields E, chemical potential μ, inverse temperature β, and relaxation time (τ). These flow parameters are chosen to demonstrate the effects of nonequilibrium situation on the BTE dynamics. In order to investigate these nonequilibrium effects, the electric field values $E_{0}$ range from 1∼$10^{6}$ V/cm. The chemical potential μ is selected to range from −1.0 to 1.0, while the inverse temperature β ranges from 1 (smooth distribution) to $10^{2}$ (sharp distribution). Three different relaxation time parameters are chosen for the extensive studies, including τ=0.01,0.1, and 1.0. A wide range of frequency values (ω) has been chosen from 0.01−20. In all the numerical simulations, a polynomial expansion of third-order accuracy is used to approximate solutions in the finite element space. All the computations are carried out on 300 grid points, and the periodic boundary conditions are enforced at the right and left boundaries. The initial condition for the distribution function is considered as Fermi-Dirac function.

### 6.1. Time Evolution of BTE Dynamics: Effects of Flow Parameters

**Figure 4:**
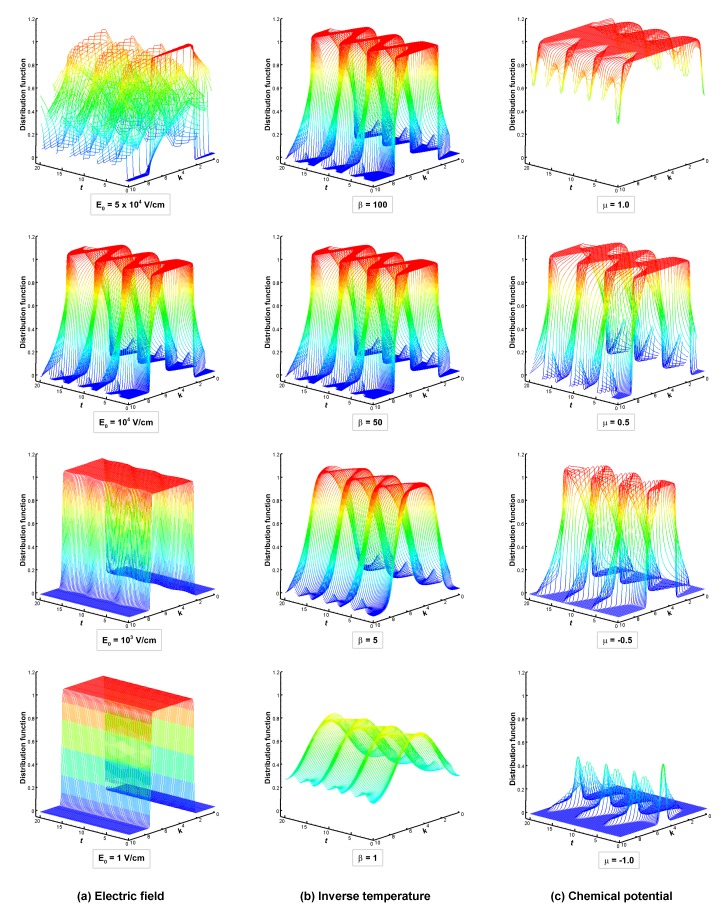
Effect of flow parameters on Boltzmann transport equation (BTE) dynamics: time evolution of distribution function at (**a**) electric field $E_{0}=1,10^{3},10^{4},5\times 10^{4}$ V/cm with τ=1.0, β=50, μ=0, and ω=1; (**b**) inverse temperature β=1,5,50,100 with $E_{0}=10^{4}$ V/cm, τ=1.0, μ=0, and ω=1; (**c**) chemical potential μ=−1.0,−0.5,0.5,1.0 with $E_{0}=10^{4}$ V/cm, τ=1.0, β=50, and ω=1.

Flow parameters—the electric field, the inverse temperature, and the chemical potential—play a critical role in ultrafast nonequilibrium electron dynamics. Therefore, we conducted a detailed investigation into the effects of these Flow parameters on BTE dynamics. To demonstrate the effects of the electric field on BTE dynamics, four different electric field values, $E_{0}=1,10^{3},10^{4}$, and $5\;\times \;10^{4}$ V/cm, are selected with the same τ=1.0, β=50, μ=0, and ω=1. Figure 4a illustrates the time evolution of the electronic distribution function for different electric fields.

At a low electric field $E_{0}=1$ V/cm, the distribution function remains close to the equilibrium state, as shown in Figure 4a. We stress that the shift of the Fermi surface (not shown), although very small, is properly reproduced by the numerics, as showed in the previous section. The steady state is reached after a time of the order of the relaxation time. The transient shows a relatively simple behavior with the distribution progressively approaching the steady-state response.

As the electric field value increases to $E_{0}=10^{3}$ V/cm, the steady-state distribution is still near to the equilibrium distribution, as shown in Figure 4a. However, the population is not a rigid shift of the Fermi surface anymore. Even at low temperatures, the Fermi edge, on top of being shifted, is also smoothened. The system is already beyond the close-to-equilibrium regime, and the electric field cannot be considered a small perturbation anymore. The transient time does not look significantly altered by the strength of the electric field.

When the electric field value increases to $E_{0}=10^{4}$ V/cm, the system enters into the dampened Bloch oscillation regime. The distribution function undergoes strong accelerations, which push electrons across the border of the Brillouin zone triggering Bloch oscillations. In Figure 4a, one can observe a sizable population traversing the border of the Brillouin zone. Due to the presence of the scattering operator however, the sharp profile of the electrons’ distribution is eventually dampened, and the system reaches a far-from-equilibrium steady state. At very high electric field value $E_{0}=5\times 10^{4}$ V/cm, the distribution undergoes faster crossing of the Brillouin zone before reaching a steady state, as shown in Figure 4a. The motion is not fully resolved in the figure since only a fraction of the computed time steps have been plotted.

To analyze the effects of the inverse temperature $\left(\beta =1/k_{B}T\right)$ on the BTE dynamics, four different inverse temperature values, β=1,5,50 and $10^{2}$, are selected with the same electric field value $E_{0}=10^{4}$ V/cm with μ=0, τ=1, and ω=1. We focus on the most interesting regime: the dampened Bloch oscillation regime shown for different inverse temperatures in Figure 4b. At higher temperatures, the electronic distribution is smooth, as shown in Figure 4b. At low temperatures, the distribution displays sharp drops at the Fermi level (in principle, it becomes discontinuous at T=0). Notice how, in spite of the almost discontinuous solution, the numerical method proved to perform well and remained stable even at very low temperature, as seen in Figure 4b.

Interestingly, one can clearly distinguish the transition between two regimes. At very high temperature, the steady-state distribution shape is strongly smoothened due to the temperature. On the other hand, at very low temperature, the steady-state solution becomes completely dominated by the electric field: as can be observed in Figure 4b, the steady-state distributions are practically independent of the initial temperature.

Further, we show the results for several positions of the chemical potential for a strong electric field. Figure 4c illustrates the effect of the chemical potential μ on the time evolution of the distribution function with $E_{0}=10^{4}$ V/cm, τ=1.0, β=50, and ω=1.0. Here, we selected different chemical potential values between −1.0 to 1.0, as shown in Figure 4c. The pictures show the transition from an electron regime to a hole regime.

### 6.2. High Field Current Density and Conductivities: The Bloch Oscillation Resonance

**Figure 5:**
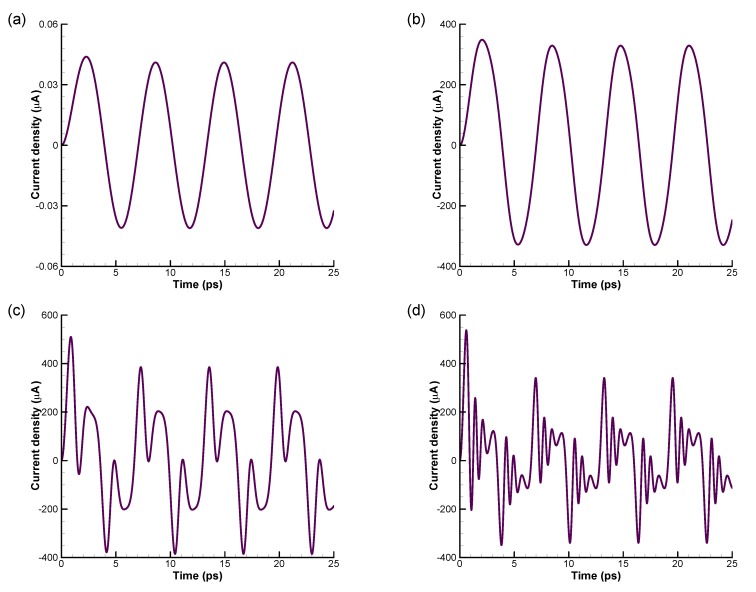
Effect of electric field on the time-dependent current density at (**a**) $E_{0}=1$ V/cm, (**b**) $E_{0}=10^{4}$ V/cm, (**c**) $E_{0}=5\times 10^{4}$ V/cm, and (**d**) $E_{0}=10^{5}$ V/cm with τ=1.0, β=50, μ=0, and ω=1.

Here, we analyze in more detail the effect of high electric fields onto the material responses, focusing on the effect on the induced current. Figure 5 illustrates the effects of electric fields on the time evolution of oscillating current density with τ=1.0, β=50, μ=0, and ω=1.0.

We first focus on the transient behavior. At a small electric field $E_{0}=1$ V/cm, the current density reaches the steady-state response after a transient of the order of few relaxation times, as shown in Figure 5a. Interestingly, during the transient, the current density value first grows above the steady-state response amplitude. This behavior is consistently observed for all the electric fields’ amplitudes.

#### 6.2.1. Frequency Dependence: The Bloch Oscillation Resonance

We now address the steady-state response, which is reached after the transient response. It can be observed that there are significant differences in time evolution of the current density with increasing electric field value. The first evident effect is the appearance of response at higher harmonics. This is expected even in the small perturbation regime, since the responses of the system at higher harmonics are proportional to higher powers of the field amplitude, and become more and more relevant at higher fields. However, there is a further effect playing an important role: the proportionality constants of the lower harmonics (conductivity) are importantly altered in high fields.

**Figure 6:**
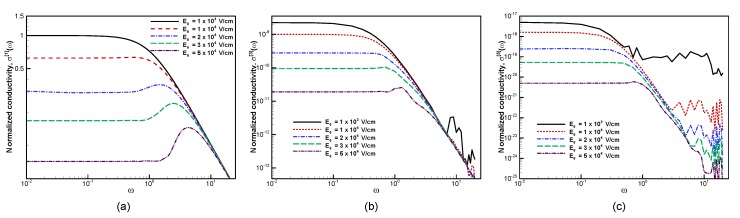
Effect of electric fields on the calculated (**a**) first order, (**b**) third-order, and (**c**) fifth-order conductivity at β=50, μ=−0.8, and τ=1. All conductivities are normalized to low-field, zero frequency conductivity. The peaks appearing at frequencies increasing with electric field are the Bloch oscillation resonances.

Figure 6 shows the frequency-dependence of the first- (conductivity), the third-, and the fifth-order responses at increasing electric fields (all the values are normalized to the low-field conductivity at zero frequency). At low fields (line corresponding to $E_{0}=10^{3}$ V/cm) the first order response is simply the conductivity reported in Figure 2a. However as the electric field is increased, the response of the material shows high field effects, that strongly depend on the frequency of the external field.

At low frequencies, the conductivity decreases (see Figure 6a) as it enters the dampened Bloch oscillation regime: a large electric field can accelerate electrons across the border of the Brillouin zone, where the electron motion inverts direction, depressing the conductivity. Conversely, the reduction in the conductivity is instead not observed at high frequencies: given the high frequency of the oscillating frequency, even in the presence of a high field, electrons still remain relatively close to the Fermi surface and keep responding in the linear regime.

The most interesting regime is between the abovementioned ones. It is easier to understand the physics behind this regime by first analyzing the dynamics of an electron in a static electric field. As an electron is accelerated across the Brillouin zone due to a static electric field, its velocity changes in time, generating a time-dependent contribution to the current which oscillates with a Bloch oscillation period

(22) $$T_{res}=\frac{\hbar \;\Delta K}{e\;E_{0}},$$

where ΔK is the size of the Brillouin zone. The reader should note how the Bloch oscillation frequency increases linearly with the electric field’s amplitude. However, at low electric fields, such response can hardly be observed since such oscillations are dampened by the scattering term. Therefore, only at sufficiently high electric fields, when the oscillation period is sufficiently shorter than the relaxation time, can these dynamics become observable. In case the external electric field oscillates, a similar dynamics is triggered. However, it is important to realize that, when doing the Fourier transform of the current, it will produce contributions at its resonance frequency and not necessarily at the frequency of the applied field. However, if the applied electric field happens to be at a frequency close or equal to the Bloch oscillation frequency, a resonance appears in the conductivity. This is clearly observed in Figure 2a, where no resonance is observed at low fields; while for increasing fields, the Bloch resonance is observed to both increase in strength and shift to higher frequencies.

The effect appears in the higher-order conductivities as well, but it can be observed to be triggered at lower external field’s frequencies. This is indeed understandable since, as mentioned, the Bloch oscillation frequency is dependent only on the amplitude of the external field and always gives a response at that frequency. When the electric field’s frequency is one third of the Bloch oscillation frequency, the third-order response in the current happens to be exactly resonant with the Bloch oscillations, giving the peak observed in Figure 2b. In the fifth-order response in Figure 6c, one can observe how the resonance is triggered at even lower frequency, which is one fifth of the Bloch oscillation frequency or the frequency at which the resonance is observed in conductivity.

As a further comment, let us highlight that in the higher-order responses, if the electric field is small, the overall high-frequency component of the current becomes too small and the computed response simply shows the numerical error.

#### 6.2.2. Chemical Potential and Temperature Dependences

**Figure 7:**
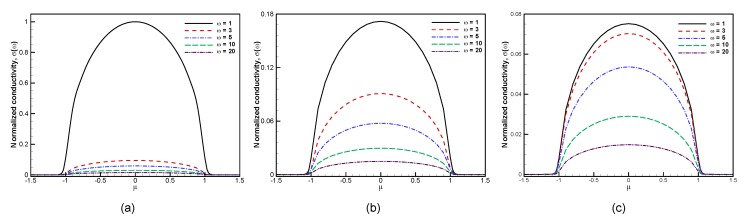
Computed electrical conductivity vs. chemical potential for various electric frequency ω=1,3,5,10,20 at (**a**) $E_{0}=10^{3}$ V/cm, (**b**) $E_{0}=10^{4}$ V/cm, and (**c**) $E_{0}=2.5\times 10^{4}$ V/cm with β=50 and τ=1.0.

Finally, we analyze how the high-field conductivity depends on the other parameters of the system. The dependence on the chemical potential in Figure 7 does not show importantly new effects, as the conductivity remains proportional to the number of free carriers (whether electrons or holes) within the band, even at high fields. The fact that low-frequency responses are more strongly suppressed at higher fields is also clearly observed.

**Figure 8:**
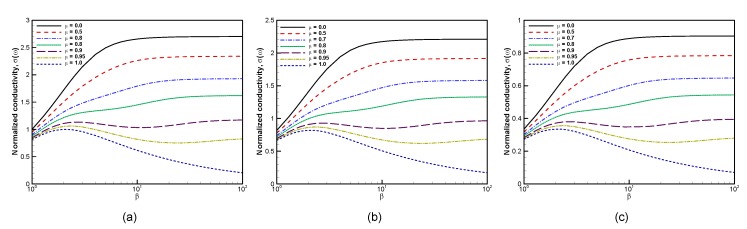
Computed electrical conductivity vs. inverse temperature for various chemical potential values μ=0.0,0.5,0.7,0.8,0.9,0.95,1.0 at (**a**) $E_{0}=10^{3}$ V/cm, (**b**) $E_{0}=10^{4}$ V/cm, and (**c**) $E_{0}=2.5\times 10^{4}$ V/cm with ω=1 and τ=1.0.

Figure 8 finally shows the dependence of the conductivity with the inverse temperature with increasing the applied electric field. Apart from a suppression of the conductivity, the overall dependence is not altered when increasing the amplitude of the electric field.

## 7. Concluding Remarks

This study focused on an investigation of the strong electric field’s effect on material responses and the Bloch oscillation resonance in high-field conductivities. For this purpose, a high-order accurate explicit modal discontinuous Galerkin is applied to study the transport quantum Boltzmann equation far from equilibrium. We first focused on the analysis of performance of the presented method by testing its precision to close-to-equilibrium conditions. We have compared the method to analytic results for the low-field steady-state regime and showed that it achieves extremely high precision, even if the method is devised for strong excitations and not optimized for extremely small deviations from equilibrium. Secondly, we have performed an extensive range of numerical simulations to investigate the effects of strong electric fields, and the dependence of the response on a range of physical parameters. In particular, we have shown how corrections to conductivity arise at high fields. This allowed us to identify a new regime, appearing at high electric fields, triggered by the resonance of typical material responses and dampened Bloch oscillations. Experimental confirmation of the appearance of such resonance requires the choice of a material with a relatively long scattering lifetime (for example, low-temperature, high-purity silicon) in order to allow the effect to be present at not too high frequencies where different contributions to conductivity start playing important roles.

## Figures and Tables

**Table 1:** Convergence study of the initial value problem.

*l*	Δk	DOF	$L^{2}-$ Error	Order	$L^{\infty }-$ Error	Order
1	2π/20	40	0.689292 $\times 10^{-1}$	−	0.416916 $\times 10^{-1}$	−
	2π/40	80	0.179328 $\times 10^{-1}$	1.93	0.106198 $\times 10^{-1}$	1.96
	2π/80	160	0.453598 $\times 10^{-2}$	1.97	0.265698 $\times 10^{-2}$	2.01
	2π/160	320	0.113801 $\times 10^{-2}$	1.98	0.663269 $\times 10^{-3}$	2.00
2	2π/20	60	0.104224 $\times 10^{-2}$	−	0.124147 $\times 10^{-2}$	−
	2π/40	120	0.130558 $\times 10^{-3}$	3.03	0.158800 $\times 10^{-3}$	2.95
	2π/80	240	0.163282 $\times 10^{-4}$	3.02	0.200245 $\times 10^{-4}$	2.98
	2π/160	480	0.204129 $\times 10^{-5}$	3.01	0.251331 $\times 10^{-5}$	2.99
3	2π/20	80	0.176257 $\times 10^{-4}$	−	0.270453 $\times 10^{-4}$	−
	2π/40	160	0.110354 $\times 10^{-5}$	4.01	0.170007 $\times 10^{-5}$	3.98
	2π/80	320	0.689895 $\times 10^{-7}$	4.03	0.106066 $\times 10^{-6}$	3.99
	2π/160	640	0.431213 $\times 10^{-8}$	4.02	0.662179 $\times 10^{-8}$	4.00
4	2π/20	100	0.252754 $\times 10^{-6}$	−	0.429430 $\times 10^{-6}$	−
	2π/40	200	0.798519 $\times 10^{-8}$	5.05	0.143225 $\times 10^{-7}$	4.92
	2π/80	400	0.246970 $\times 10^{-9}$	5.01	0.444279 $\times 10^{-9}$	4.99
	2π/160	800	0.771945 $\times 10^{-11}$	5.03	0.138960 $\times 10^{-10}$	5.01
